# 3-(4-Bromo­phen­yl)-1-(4-chloro­benz­yl)-1*H*-pyrazole-5-carbaldehyde

**DOI:** 10.1107/S1600536812027298

**Published:** 2012-06-23

**Authors:** Fu-Rong Li, Yu-Juan Zhang, Feng-Guang Guo, Gui-Yun Duan, Yan-Qing Ge

**Affiliations:** a Taishan Medical University, Tai an 271016, People’s Republic of China

## Abstract

The title compound, C_17_H_12_BrClN_2_O, was synthesized by oxidation of [3-(4-bromo­phen­yl)-1-(4-chloro­benz­yl)-1*H*-pyrazol-5-yl]methanol under mild conditions. The pyrazole ring makes dihedral angles of 3.29 (9) and 74.91 (4)°, respectively, with the bromo­phenyl and chloro­phenyl rings.

## Related literature

For applications of nitro­gen-containing heterocyclic compounds in the agrochemical and pharmaceutical fields, see: Ge *et al.* (2007 ▶, 2009 ▶, 2011 ▶). For the biological activity of some pyrazole derivatives belonging to this class of compounds, see: Xia *et al.* (2007 ▶). For a related compound, see: Hao *et al.* (2012 ▶).
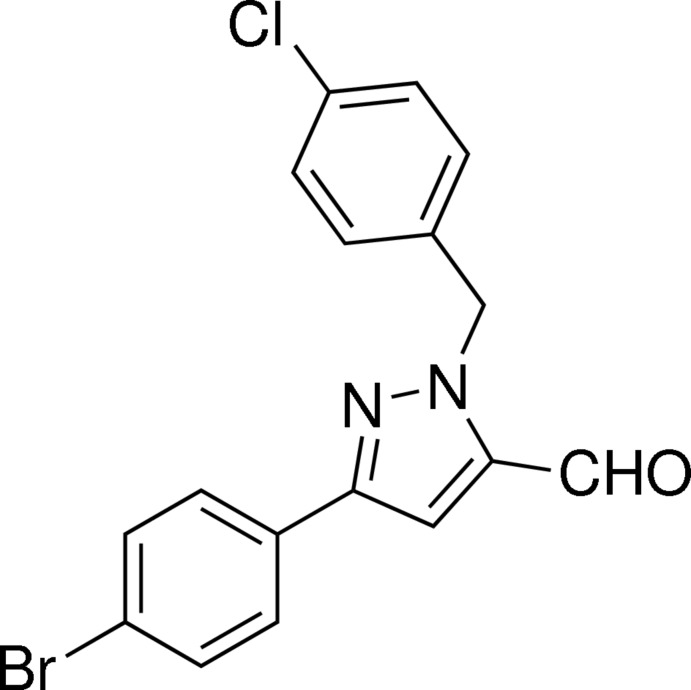


## Experimental

### 

#### Crystal data

C_17_H_12_BrClN_2_O
*M* *_r_* = 375.65
Triclinic, 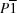

*a* = 6.759 (5) Å
*b* = 10.061 (5) Å
*c* = 12.263 (5) Å
α = 109.080 (5)°
β = 94.521 (5)°
γ = 93.098 (5)°
*V* = 782.8 (8) Å^3^
*Z* = 2
Mo *K*α radiation
μ = 2.80 mm^−1^
*T* = 293 K
0.18 × 0.15 × 0.14 mm

#### Data collection

Bruker SMART APEX CCD area-detector diffractometer
Absorption correction: multi-scan (*SADABS*; Bruker, 2005 ▶) *T*_min_ = 0.860, *T*_max_ = 0.891
4563 measured reflections
3151 independent reflections
2410 reflections with *I* > 2σ(*I*)
*R*_int_ = 0.013

#### Refinement

*R*[*F* ^2^ > 2σ(*F* ^2^)] = 0.036
*wR*(*F* ^2^) = 0.104
*S* = 1.05
3151 reflections
200 parameters
H-atom parameters constrained
Δρ_max_ = 0.50 e Å^−3^
Δρ_min_ = −0.43 e Å^−3^

### 

Data collection: *SMART* (Bruker, 2005 ▶); cell refinement: *SAINT* (Bruker, 2005 ▶); data reduction: *SAINT*; program(s) used to solve structure: *SHELXS97* (Sheldrick, 2008 ▶); program(s) used to refine structure: *SHELXL97* (Sheldrick, 2008 ▶); molecular graphics: *XP* in *SHELXTL* (Sheldrick, 2008 ▶); software used to prepare material for publication: *SHELXL97*.

## Supplementary Material

Crystal structure: contains datablock(s) I, global. DOI: 10.1107/S1600536812027298/bh2439sup1.cif

Structure factors: contains datablock(s) I. DOI: 10.1107/S1600536812027298/bh2439Isup2.hkl

Supplementary material file. DOI: 10.1107/S1600536812027298/bh2439Isup3.cml

Additional supplementary materials:  crystallographic information; 3D view; checkCIF report

## Figures and Tables

**Fig. 1.**
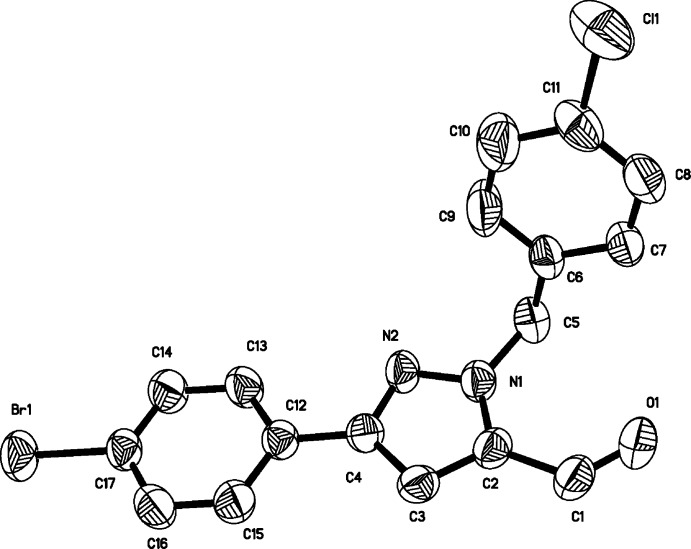
The molecular structure of the title compound, showing displacement ellipsoids drawn at the 50% probability level. H atoms have been omitted.

## supplementary crystallographic information

### Comment

Synthesis of nitrogen-containing heterocyclic compounds has been a subject of great interest due to the wide application in agrochemical and pharmaceutical fields (Ge *et al.* 2007, 2009, 2011). Some pyrazole derivatives which belong to this category have been of interest for their biological activities (Xia *et al.*, 2007). Considerable efforts have been devoted to the development of novel pyrazole compounds. The title compound (Fig. 1) is a new pyrazole derivative, which was synthesized in order to study and compare its biological properties with other related compounds (Xia *et al.*, 2007). The title compound was screened for anticancer activities and found to be inactive. We report here the crystal structure of the title compound. The pyrazole ring makes dihedral angles of 3.29 (9) and 74.91 (4)°, respectively, with the bromophenyl and chlorophenyl rings. This conformation is close to that found in a related pyrazole derivative (Hao *et al.*, 2012).

### Experimental

A mixture of (3-(4-bromophenyl)-1-(4-chlorobenzyl)-1*H*-pyrazol-5-yl)methanol (0.02 mol) and PCC (0.06 mol) in DMF (50 ml) was stirred for 3 h. After the starting material was consumed (monitored by TLC), the reaction mixture was poured into water (100 ml) and extracted with dichloromethane. The organic extracts were washed with water, dried, filtered and concentrated. The final product was isolated by column chromatography on silica gel (yield 72%). Crystals of the title compound suitable for X-ray diffraction were obtained by allowing a refluxed solution of the product in ethyl acetate (0.10 *M*) to cool slowly to room temperature (without temperature control) and allowing the solvent to evaporate for 3 days.

### Refinement

All H atoms were placed in geometrically calculated positions and refined using a riding model with C—H = 0.97 Å (for the CH_2_ group) and 0.93 Å (for aromatic CH); their isotropic displacement parameters were set to 1.2 times the equivalent displacement parameter of their parent atoms.

### Crystal data

**Table:**

C_17_H_12_BrClN_2_O	*Z* = 2
*M**_r_* = 375.65	*F*(000) = 376
Triclinic, *P*1	*D*_x_ = 1.594 Mg m^−^^3^
Hall symbol: -P 1	Mo *K*α radiation, λ = 0.71069 Å
*a* = 6.759 (5) Å	Cell parameters from 5043 reflections
*b* = 10.061 (5) Å	θ = 0.9–28.3°
*c* = 12.263 (5) Å	µ = 2.80 mm^−^^1^
α = 109.080 (5)°	*T* = 293 K
β = 94.521 (5)°	Block, colourless
γ = 93.098 (5)°	0.18 × 0.15 × 0.14 mm
*V* = 782.8 (8) Å^3^	

### Data collection

**Table:**

Bruker SMART APEX CCD area-detector diffractometer	3151 independent reflections
Radiation source: fine-focus sealed tube	2410 reflections with *I* > 2σ(*I*)
Graphite monochromator	*R*_int_ = 0.013
φ and ω scans	θ_max_ = 26.4°, θ_min_ = 2.3°
Absorption correction: multi-scan (*SADABS*; Bruker, 2005)	*h* = −8→4
*T*_min_ = 0.860, *T*_max_ = 0.891	*k* = −12→11
4563 measured reflections	*l* = −13→15

### Refinement

**Table:**

Refinement on *F*^2^	Secondary atom site location: difference Fourier map
Least-squares matrix: full	Hydrogen site location: inferred from neighbouring sites
*R*[*F*^2^ > 2σ(*F*^2^)] = 0.036	H-atom parameters constrained
*wR*(*F*^2^) = 0.104	*w* = 1/[σ^2^(*F*_o_^2^) + (0.0602*P*)^2^ + 0.068*P*] where *P* = (*F*_o_^2^ + 2*F*_c_^2^)/3
*S* = 1.05	(Δ/σ)_max_ = 0.001
3151 reflections	Δρ_max_ = 0.50 e Å^−^^3^
200 parameters	Δρ_min_ = −0.43 e Å^−^^3^
0 restraints	Extinction correction: *SHELXL97* (Sheldrick, 2008), Fc^*^=kFc[1+0.001xFc^2^λ^3^/sin(2θ)]^-1/4^
0 constraints	Extinction coefficient: 0.019 (3)
Primary atom site location: structure-invariant direct methods	

### Fractional atomic coordinates and isotropic or equivalent isotropic displacement parameters (Å^2^)

**Table:**

	*x*	*y*	*z*	*U*_iso_*/*U*_eq_	
Br1	1.33740 (5)	−0.03239 (4)	−0.36464 (3)	0.07272 (18)	
C1	0.2091 (4)	0.4007 (3)	0.0013 (3)	0.0622 (7)	
H1	0.1666	0.4399	−0.0546	0.075*	
C2	0.3841 (4)	0.3206 (3)	−0.0184 (2)	0.0488 (6)	
C3	0.4976 (4)	0.2937 (3)	−0.1103 (2)	0.0486 (6)	
H3	0.4800	0.3243	−0.1740	0.058*	
C4	0.6443 (4)	0.2110 (2)	−0.0884 (2)	0.0439 (6)	
C5	0.4145 (4)	0.2593 (3)	0.1680 (2)	0.0529 (6)	
H5A	0.4587	0.1766	0.1837	0.063*	
H5B	0.2711	0.2569	0.1693	0.063*	
C6	0.5106 (4)	0.3908 (3)	0.2619 (2)	0.0476 (6)	
C7	0.3960 (4)	0.4906 (3)	0.3274 (2)	0.0533 (7)	
H7	0.2582	0.4783	0.3110	0.064*	
C8	0.4806 (5)	0.6079 (3)	0.4166 (2)	0.0588 (7)	
H8	0.4013	0.6738	0.4601	0.071*	
C9	0.7135 (5)	0.4130 (4)	0.2855 (3)	0.0772 (10)	
H9	0.7940	0.3483	0.2415	0.093*	
C10	0.7995 (5)	0.5311 (4)	0.3743 (3)	0.0867 (11)	
H10	0.9374	0.5459	0.3893	0.104*	
C11	0.6828 (5)	0.6255 (3)	0.4398 (2)	0.0606 (8)	
C12	0.8084 (4)	0.1521 (3)	−0.1560 (2)	0.0449 (6)	
C13	0.9331 (4)	0.0666 (3)	−0.1209 (2)	0.0580 (7)	
H13	0.9110	0.0459	−0.0541	0.070*	
C14	1.0906 (5)	0.0105 (3)	−0.1818 (3)	0.0624 (8)	
H14	1.1735	−0.0465	−0.1562	0.075*	
C15	0.8436 (5)	0.1785 (3)	−0.2571 (3)	0.0591 (7)	
H15	0.7611	0.2351	−0.2835	0.071*	
C16	0.9981 (5)	0.1229 (3)	−0.3193 (3)	0.0616 (7)	
H16	1.0186	0.1408	−0.3875	0.074*	
C17	1.1211 (4)	0.0411 (3)	−0.2803 (2)	0.0497 (6)	
Cl1	0.79191 (16)	0.76908 (10)	0.55587 (7)	0.0864 (3)	
N1	0.4642 (3)	0.2545 (2)	0.05291 (18)	0.0478 (5)	
N2	0.6237 (3)	0.1883 (2)	0.01222 (19)	0.0482 (5)	
O1	0.1149 (3)	0.4203 (3)	0.0836 (2)	0.0785 (6)	

### Atomic displacement parameters (Å^2^)

**Table:**

	*U*^11^	*U*^22^	*U*^33^	*U*^12^	*U*^13^	*U*^23^
Br1	0.0582 (2)	0.0828 (3)	0.0653 (2)	0.01627 (16)	0.02256 (15)	0.00339 (16)
C1	0.0485 (16)	0.0687 (18)	0.0609 (17)	0.0207 (14)	−0.0006 (14)	0.0088 (14)
C2	0.0431 (14)	0.0466 (13)	0.0507 (14)	0.0101 (11)	0.0009 (11)	0.0079 (11)
C3	0.0502 (15)	0.0489 (14)	0.0447 (14)	0.0116 (12)	−0.0003 (11)	0.0127 (11)
C4	0.0457 (14)	0.0406 (12)	0.0438 (13)	0.0081 (10)	0.0052 (11)	0.0110 (10)
C5	0.0530 (16)	0.0523 (15)	0.0565 (16)	0.0091 (12)	0.0201 (12)	0.0183 (12)
C6	0.0464 (15)	0.0530 (14)	0.0476 (14)	0.0083 (11)	0.0178 (11)	0.0186 (12)
C7	0.0535 (16)	0.0626 (17)	0.0469 (14)	0.0163 (13)	0.0160 (12)	0.0181 (13)
C8	0.071 (2)	0.0601 (17)	0.0475 (15)	0.0200 (14)	0.0202 (13)	0.0154 (13)
C9	0.0508 (18)	0.079 (2)	0.080 (2)	0.0106 (15)	0.0246 (16)	−0.0071 (17)
C10	0.0503 (19)	0.100 (3)	0.088 (3)	−0.0059 (18)	0.0204 (17)	−0.001 (2)
C11	0.076 (2)	0.0576 (16)	0.0471 (15)	−0.0060 (14)	0.0220 (14)	0.0137 (13)
C12	0.0445 (14)	0.0426 (13)	0.0452 (13)	0.0060 (11)	0.0065 (11)	0.0107 (11)
C13	0.0630 (18)	0.0687 (18)	0.0516 (15)	0.0275 (14)	0.0175 (13)	0.0263 (14)
C14	0.0639 (18)	0.0686 (18)	0.0604 (17)	0.0303 (15)	0.0158 (14)	0.0232 (15)
C15	0.0572 (17)	0.0703 (18)	0.0621 (17)	0.0221 (14)	0.0133 (14)	0.0345 (15)
C16	0.0671 (19)	0.0727 (19)	0.0515 (16)	0.0130 (15)	0.0179 (14)	0.0254 (14)
C17	0.0448 (14)	0.0493 (14)	0.0469 (14)	0.0064 (11)	0.0103 (11)	0.0035 (11)
Cl1	0.1054 (7)	0.0785 (6)	0.0600 (5)	−0.0247 (5)	0.0211 (4)	0.0047 (4)
N1	0.0454 (12)	0.0472 (11)	0.0491 (12)	0.0106 (9)	0.0109 (9)	0.0115 (10)
N2	0.0470 (12)	0.0473 (12)	0.0527 (13)	0.0162 (9)	0.0148 (10)	0.0156 (10)
O1	0.0548 (13)	0.1008 (17)	0.0737 (15)	0.0337 (12)	0.0132 (11)	0.0150 (13)

### Geometric parameters (Å, º)

**Table:**

Br1—C17	1.900 (3)	C8—C11	1.363 (4)
C1—O1	1.203 (4)	C8—H8	0.9300
C1—C2	1.459 (4)	C9—C10	1.384 (5)
C1—H1	0.9300	C9—H9	0.9300
C2—N1	1.358 (3)	C10—C11	1.361 (5)
C2—C3	1.376 (4)	C10—H10	0.9300
C3—C4	1.393 (4)	C11—Cl1	1.741 (3)
C3—H3	0.9300	C12—C13	1.378 (4)
C4—N2	1.342 (3)	C12—C15	1.385 (4)
C4—C12	1.471 (4)	C13—C14	1.387 (4)
C5—N1	1.462 (3)	C13—H13	0.9300
C5—C6	1.514 (4)	C14—C17	1.368 (4)
C5—H5A	0.9700	C14—H14	0.9300
C5—H5B	0.9700	C15—C16	1.377 (4)
C6—C9	1.370 (4)	C15—H15	0.9300
C6—C7	1.382 (4)	C16—C17	1.365 (4)
C7—C8	1.379 (4)	C16—H16	0.9300
C7—H7	0.9300	N1—N2	1.342 (3)
			
O1—C1—C2	125.6 (3)	C10—C9—H9	119.8
O1—C1—H1	117.2	C11—C10—C9	120.2 (3)
C2—C1—H1	117.2	C11—C10—H10	119.9
N1—C2—C3	106.4 (2)	C9—C10—H10	119.9
N1—C2—C1	125.1 (3)	C10—C11—C8	120.7 (3)
C3—C2—C1	128.5 (3)	C10—C11—Cl1	119.8 (3)
C2—C3—C4	105.7 (2)	C8—C11—Cl1	119.5 (2)
C2—C3—H3	127.1	C13—C12—C15	117.2 (2)
C4—C3—H3	127.1	C13—C12—C4	120.7 (2)
N2—C4—C3	110.4 (2)	C15—C12—C4	122.1 (2)
N2—C4—C12	119.5 (2)	C12—C13—C14	122.3 (3)
C3—C4—C12	130.0 (2)	C12—C13—H13	118.9
N1—C5—C6	111.9 (2)	C14—C13—H13	118.9
N1—C5—H5A	109.2	C17—C14—C13	118.4 (3)
C6—C5—H5A	109.2	C17—C14—H14	120.8
N1—C5—H5B	109.2	C13—C14—H14	120.8
C6—C5—H5B	109.2	C16—C15—C12	121.4 (3)
H5A—C5—H5B	107.9	C16—C15—H15	119.3
C9—C6—C7	118.2 (3)	C12—C15—H15	119.3
C9—C6—C5	120.9 (2)	C17—C16—C15	119.6 (3)
C7—C6—C5	120.9 (3)	C17—C16—H16	120.2
C8—C7—C6	121.7 (3)	C15—C16—H16	120.2
C8—C7—H7	119.2	C16—C17—C14	121.1 (3)
C6—C7—H7	119.2	C16—C17—Br1	119.5 (2)
C11—C8—C7	118.8 (3)	C14—C17—Br1	119.4 (2)
C11—C8—H8	120.6	N2—N1—C2	111.8 (2)
C7—C8—H8	120.6	N2—N1—C5	118.3 (2)
C6—C9—C10	120.4 (3)	C2—N1—C5	129.5 (2)
C6—C9—H9	119.8	N1—N2—C4	105.6 (2)
